# Toxicological investigation of acute and chronic treatment with *Gnidia stenophylla* Gilg root extract on some blood parameters and histopathology of spleen, liver and kidney in mice

**DOI:** 10.1186/s13104-017-2964-3

**Published:** 2017-11-28

**Authors:** Tilahun Alemayehu Nigatu, Mekbeb Afework, Kelbessa Urga, Wondwossen Ergete, Eyasu Makonnen

**Affiliations:** 1 Anatomy Course Team, Department of Biomedical Sciences, Institute of Health, Jimma University, P.O.Box 378, Jimma, Ethiopia; 2 Department of Anatomy, College of Health Sciences, Addis Ababa University, Addis Ababa, Ethiopia; 3 Ethiopian Public Health Institute, Traditional and Modern Medicine, Vaccines Directorate, Addis Ababa, Ethiopia; 4 Department of Pathology, College of Health Sciences, Addis Ababa University, Addis Ababa, Ethiopia; 5 Department of Pharmacology, College of Health Sciences, Addis Ababa University, Addis Ababa, Ethiopia

**Keywords:** *Gnidia stenophylla* Gilg, Aqueous root extract, Acute toxicity, Chronic toxicity, Hematology, Histopathology, Hepatotoxicity

## Abstract

**Background:**

In southeast Ethiopia, people locally use the roots of *Gnidia stenophylla* Gilg (Thymelaeaceae) to cure malaria and other diseases with no literature evidence substantiating its safety. The aim of this study was, therefore, to investigate the safety of the aqueous root extract of *G. stenophylla* after acute (single dose) and repeated sub chronic oral administration in mice.

**Results:**

A single oral administration of the extract at 500, 1000, 2000 and 4000 mg/kg body weight did not induce any behavioral change and mortality in both sexes. The oral LD_50_ of the extract was found to be above 6000 mg/kg body weight in mice. Chronic treatment with the extract for 13 weeks did not induce any sign of illness and/or death and had no adverse effect on the body weight. Dose-related elevations of erythrocytes, hematocrit, hemoglobin, platelets and neutrophils differential and significant decrease in the number of lymphocyte were observed. Liver sections of mice treated with 800 mg/kg body weight, revealed mild inflammations around the portal triads and central veins; whereas the spleen and kidneys appeared normal with no detectable gross morphological and histopathological alteration at both doses.

**Conclusions:**

The results of this study revealed that aqueous root extract of *G. stenophylla* Gilg at antimalarial dose is safe even when taken for a longer period. At a higher dose, the extract may have a potential to increase some hematological indices but may induce mild hepatotoxicity as a side effect.

**Electronic supplementary material:**

The online version of this article (10.1186/s13104-017-2964-3) contains supplementary material, which is available to authorized users.

## Background

Traditional medicine is an ancient medical practice that is still widely used in prevention and treatment of various health problems worldwide, including Ethiopia [1–4]. There is a growing recognition that knowledge of traditional medicine is important not only for its potential as therapeutic drugs but also for its socioeconomic and cultural components [5–11]. Many of the modern pharmaceuticals are derived from medicinal herbs. Some of these include digitalis, a heart medication, derived from the Foxglove plant; salicylic acid, the source of aspirin, from Willow bark; and taxol for treating ovarian cancer, from the Pacific Yew tree [9]. As mentioned above, quinine and atremisinine are also well known antimalarial drugs derived from the bark of *Cinchona* tree and *Atremisia annua*, respectively [12].

Although many health problems have been treated by using medicinal plants, acute and chronic phytotoxic effects of some plants are also reported [13–16]. Therefore, scientific approach needs to be applied towards the safety and effectiveness of traditional plants in managing ailments. WHO [8] emphasizes the importance of scientific investigations into indigenous herbal medicines and incorporated acute and chronic toxicological studies as part of the safety assessment of herbal products.

*Gnidia stenophylla* Gilg (GSG) (family-*Thymelaeaceae*), commonly known as “*Kataricha*” in Afan Oromo is one of the most widely used medicinal plants [17, 18]. The plant is a woody-based perennial herb (about 20–60 cm high) found growing naturally in Ethiopia and in other tropical and sub-tropical African countries [19, 20]. It appears with numerous erect and slender stems, arising from a thick rhizome; sessile or sub sessile needle-like leaves and yellowish white flowers.

The medicinal properties of *Gnidia* species are attributed mainly to their roots [20–22]. In Ethiopia, aqueous preparations of the plant root are used for treating malaria and its associated symptoms [23]. The root decoction is a traditional remedy for gonorrhea, syphilis, leprosy, ascaris, rabies, heart pain and rheumatic pain [24–26]. Paste of root powder and honey is employed as a remedy for breast cancer and/or tumors. Unfortunately, the scientific data to support these claims are still scarce.

Root extracts of *Gnidia* species are known to contain chemical constituents such as diterpenes (gnidicin, gnididin, and gniditrin), daphnetin, daphnenin and coumarin, which are thought to account for many of their presumed therapeutic effects [20, 21, 24]. The diterpenes such as gnidiglaucin (isolated from *G. glauca*) and gnidilatidin (from *G. latifolia*) are believed to have excellent features as anticancer agents [20]. As per reports of Kalauni et al. [27], plant extracts with diterpenes as their active ingredient exhibit high antimalarial activity. Furthermore, mezercin (antileukaemic agent) and gnidicoumarin have been isolated from *G. lamprantha* [21]. The daphnetin and its derivative, daphnenin are thought to have antibacterial (especially against gram-positive bacteria) activity [20]. Moreover, Ferrari et al. [28] isolated several flavonol glycosides including kaempferol-3-O-glucoside, yuankanin and manniflavanone from the methanol extract of the aerial parts of *G. involucrata*.

The traditional claims of the *Gnidia* species for treatment of malaria has been confirmed by a number of pharmacological studies. Despite a wide range of traditional uses of *Gnidia* species as a whole and GSG in particular as herbal remedies in treating malaria and many other health problems, only a few studies have investigated their potential toxic effect [29]. *G. involucrata* and *G. latifolia* are known to cause enteritis, nephritis, cardiomyopathy and blistering of skin in humans [24, 25]. Another study done by Kiptoon et al. [25] reported general atrophy of the body fat and accumulation of excess fluid in the body cavities of cattle poisoned with *G. latifolia*. Marked lymphocytopaenia and lymphocytic degeneration in lymph nodes and spleen are also reported. Documentation on the poisonous plants of Ethiopia has shown that some *Gnidia* species are poisonous to humans and animals [29]. Nevertheless, there is no scientific report on the safety of using GSG as a herbal remedy. The current study is, therefore, aimed at assessing the safety of GSG by investigating any possible acute and chronic toxicity of this plant extract.

## Methods

### Collection and processing of plant material

The underground roots of GSG were freshly collected from Bale, Dello Menna area, near Melka-Cira River about 550 km Southeast of Addis Ababa. The plant material was authenticated by a taxonomist from the Traditional and Modern Medicine, Drug Research Directorate (TMMDRD) of the Ethiopia Public Health Institute (EPHI), Addis Ababa. For reference, a voucher specimen (No. 001-2009) of the entire plant was deposited at the herbarium of TMMDRD.

Ethnomedical information about the plant was obtained from publications of Assefa et al. [23] and from traditional healers in the area. The collected fresh roots were processed and extracted at the Phytochemical Laboratory of TMMDRD, EPHI. The roots were rinsed with tap water to clean off the extraneous materials and air-dried at room temperature. The dried roots were then cut into pieces and ground to fine powder. The powdered substance was then weighed using an analytical balance and stored at room temperature (22 ± 2 °C) until extraction.

### Plant material extraction

Aqueous extract of GSG root powder was used for these studies. 200 g of the powdered material of GSG roots was cold macerated according to Debella [30] with 1000 ml of double distilled deioniozed water at room temperature and agitated thoroughly overnight using an Orbital Shaker. On the next day, the mixture was filtered using Whatmann filter paper (pore size of 15.0 cm). The filtrate was placed in petri-dishes, concentrated and stored in a refrigerator at 4 °C. The mixture was then freeze-dried at 5000 mbr and − 57 °C using a lyophilizer (Labconco, Inc, USA), yielding a dark-brown amorphous crude extract. The resulting crude extract was weighed and the percentage yield was expressed as the total mass of dry powder. Extraction was repeated five times and gave average percentage yield was 4.1%. The crude extract was then kept in sealed plastic vials and stored in desiccators at room temperature till further use. For preparation of tested doses, appropriate amount of this crude extract was weighed, and dissolved in 3–5 ml distilled water immediately before administration.

### Experimental animals

A total of seventy-two adult male and female Swiss albino mice (*Mus musculus*), 6–7 weeks old weighing 20–25 g, were used in this study. All the mice were obtained from the Animal House of the TMMDRD, Addis Ababa. The animals were housed in rectangular polyacrylic cages with dust-free paddy husk as bedding material. Throughout the study period, the male and female mice were kept in separate cages to avoid breeding, and maintained under constant laboratory conditions of temperature (22 ± 2 °C) with 12 h light/dark cycle. All animals were allowed free access to standard pellet diet and clean tap water ad libitum except when starvation was otherwise needed. The food and water was changed daily and the cages were cleaned and the husk changed every 3 days. All mice were apparently healthy. An acclimatization period of 7 days was allowed before experimentation, in order to minimize any non-specific stress as suggested by different scholars in various similar studies [31–34].

All toxicity studies involving the experimental animals were conducted in compliance with general guidelines for methodologies on research and evaluation of traditional medicine as promulgated by WHO [8]. The experimental design involving the mice as well as its subsequent research report was prepared in line with all the recommendations provided in the Animal Research: Reporting of In Vivo Experiments (ARRIVE) guidelines published online in PLOS Biology [31].

### Acute toxicity study

The methods used in similar studies of Alebachew et al. [32], Kebele et al. [33] and Pieme et al. [34], were modified to evaluate the acute toxicity of oral administration of the aqueous root extract of GSG using a total of 42 adult Swiss albino mice. Prior to experimentation, these mice were starved of food for 18 h but allowed free access to drinking water. Single oral doses (500, 1000, 2000, 4000, 5000 or 6000 mg/kg body weight) of the extract were administered to six groups of mice (three males and three females each) to determine the oral LD_50_ of the extract. Control group (three male and three female mice) was given only the vehicle (distilled water). The animals were kept under observation for 72 h post-treatment in order to check for any behavioral or clinical manifestations of oral acute toxicity, such as excitement, sleep, altered feeding, vomiting, diarrhea and ataxia. At the end of the 72 h period, the mice which received the highest dose (6000 mg/kg b.w) were sacrificed and post-mortem gross observation was performed on their internal organs, namely stomach, small and large intestines, liver, kidneys and spleen.

### Chronic toxicity study

#### Selection of doses and dosing times

This experiment was conducted to investigate the effect of treatment with GSG for 13 weeks on general health, body weight and wet organ-weight as well as on blood parameters, and spleen, liver and kidney tissues. For this study, thirty male and female albino mice were used. At the commencement of the experiment, they were randomly divided into three groups (I–III) of ten animals, five males and five females each. Animals in each group were treated with aqueous root extract of GSG for 13 weeks (95 days) at different doses as follows: Group I served as a control group and received 0.5 ml/mouse distilled water orally, once a day at 24 h intervals, throughout the study period. Groups II and III were orally administered single daily doses of 400 and 800 mg/kg b.w/day of the extract respectively in 0.5 ml distilled water for 13 weeks. The tested doses were selected based on the findings of Assefa et al. [23], which reported 400 mg/kg b.w of aqueous extract of GSG roots as effective dose for in vivo antiplasmodial activity. The actual dose of the plant extract corresponding to each group was calculated based on body weight. The extract was freshly dissolved in 5 ml (0.5 ml/mouse) distilled water immediately before administration. Both the extract and the vehicle were administered orally, in a way used by the traditional healers, using a bucco-gastric cannula mounted on a 5 ml syringe.

During the whole period of the study, the animals in all groups were regularly monitored for any behavioral changes or any clinical symptoms of toxicity, such as vomiting, diarrhea, ataxia and death. As suggested by WHO [8], body weight of each mouse was measured before the start of drug administration, once a week throughout the study period, and at the end of the experiment using a semi-microbalance sensitive to 0.001 g. The body weight was recorded at the beginning of the experiment (on day 1) immediately before administration of the first dose and was considered as initial weight, while the weight taken at the end of the experiment (on day 95) immediately after the last dose administration was considered as the final body weight.

#### Specimen collection

At the end of the 13 weeks experiment, on day 95, the mice in each group were anaesthetized with diethyl ether. Blood samples were collected through a cardiac puncture using a gauge needle mounted on a 3 ml syringe. 1 ml of blood from each mouse was withdrawn and placed in sample tubes containing anti-coagulant, ethylene diamine tetra-acetic acid (EDTA) and used for determination of hematological parameters. Another 1 ml of blood was collected into sample tubes without anticoagulant and allowed to clot for 3 h. The coagulated blood was centrifuged for 5 min at 2600 revolution per minute. The sera obtained was separated with Pasteur micropipette and transferred to sterile serum bottles and used for biochemical assay.

After blood collection, all mice in the control and treated groups were sacrificed by cervical dislocation. The abdominal cavity was opened and spleen, liver and right kidney were removed from each animal and blotted on filter paper. Then each of these organs was quickly weighed on a semi-microbalance sensitive to 0.001 g and data were normalized and expressed as per 100 g body weight to obtain relative wet organ weight of each animal. After rinsing in normal saline, sections were taken from each of the harvested organs. The two coronal halves of the right kidney as well as tissue samples dissected out in block from the spleen and right lobe of the liver were placed in pre-labeled sample bottles containing fixative and used for histopathological studies. Selection to use the right lobe of the liver and the right kidney was random.

#### Hematological and biochemical analyses

Both hematological and serum biochemical assays were conducted at core laboratories of TMMDRD, Addis Ababa using standard procedures. Hematological parameters including total count of red blood cells (RBC), hemoglobin concentration (HGB), hematocrit (HCT), red cell distribution width (RDW), platelet count (PLT), total white blood cells count (WBC), and differential count of each of the leukocytes were measured in an automatic hematology analyzer, Cell-DYN-3700 (Abbott Diagnostic Division, USA). Moreover, red cell indices such as mean corpuscular volume (MCV), mean corpuscular hemoglobin (MCH) and mean corpuscular hemoglobin concentration (MCHC) were analyzed with the automatic analyzer. Similarly, serum biochemical parameters including total protein, alkaline phosphatase (ALP), alanine aminotransferase (ALT), aspartate aminotransferase (AST), urea, creatinine and cancer antigen 125 (CA-125) were determined by standard techniques in clinical chemistry analyzer, Human star 80 (Human GmbH, Germany).

#### Tissue processing, staining and histopathological evaluations

For histopathological studies under light microscopy, tissue samples taken at autopsy were processed in Histology Laboratory, Department of Anatomy, AAU. Each of the tissue samples taken from the spleen, liver and kidney were immersion-fixed separately in 10% neutral buffered formalin at room temperature. The specimens were left in the fixatives for 24 h. Following fixation, the tissues were rinsed in running water, dehydrated by immersing in ascending grades of ethyl alcohol, cleared in two changes of absolute xylene, impregnated with molten paraffin wax in hot oven, and embedded in paraffin blocks at room temperature.

The paraffin blocks were sectioned with a Leica Rotary Microtome (Leica Rm 2125RT, Model Rm2125, China) at 5–6 µ thickness. Ribbons containing every 8th to 10th sections were collected and gently floated on a tissue flotation bath at 40 °C and picked up on glass microscopic slides. Before staining, the tissue sections were deparaffinized by xylene and hydrated with ethanol. After rinsing the slides in distilled water, sections were stained regressively with Harris’ hematoxylin for 10 min. The sections were washed in tap water and dipped into 1% acid alcohol for differentiation and to remove excess stain. The sections were rinsed briefly in running tap water to remove excess acid and halt destain. The slides were then placed in saturated sodium bicarbonate solution for 3 min, and counterstained in 1% alcoholic eosin for 1 min. The H & E stained sections were dehydrated by increasing concentration of ethanol and cleared in xylene and were mounted using DPX mountant and glass cover slips.

Microscopic slides were examined under compound light microscope. Tissue sections from the treated groups were evaluated for any evidence of histopathological changes with respect to those of the controls. Photomicrographs of selected slides of each of the organs under study were taken using digital Photo camera mounted on a binocular compound microscope (Axiostar MWIB, US).

### Statistical analysis

Data obtained from the experiments were analyzed statistically on SPSS 17.0 computer software package. Data for body weight, food intake, absolute and relative organ weights as well as data for hematological and biochemical indices were presented as mean ± standard error of mean (S.E.M). Differences between the treatment and control groups were compared using one-way analysis of variance (ANOVA), followed by Dunnett’s *t* test to determine their level of significance. Post-hoc multiple range tests for high significant differences were also employed to compare between the treatment groups. Differences between the various groups were considered statistically significant at P < 0.05.

## Results

### Acute toxicity study

Oral administrations of a single dose of the extract at 500, 1000, 2000 and 4000 mg/kg b.w did not produce any mortality in both male and female mice during the 72 h observation period (Table 1). Moreover, there were no overt signs and symptoms of toxicity in these groups except weakness, low locomotor activity and piloerection seen in a few animals. During the 3 days of the observation period, these signs and symptoms completely disappeared, and all the animals treated at these doses became normal and survived beyond the observation period. However, the mice treated with extract at 5000 and 6000 mg/kg b.w showed some mild symptoms of toxicity within 1–2 h of the extract administration. These included weakness, low locomotor activity, tremor (shivering), decreased feed intake and piloerection in both male and female mice. One male mouse died among the 5000 mg/kg extract treated group, while two (one male and one female) died among the 6000 mg/kg treated mice.

**Table 1:** Effect of acute oral treatment with GSG aqueous root extract on mice

Group	Dose (mg/kg body weight)	Number of mice used per dose	Number of mice died within 72 h	% of mortality
1	Control (distilled water)	6	0	0
2	500	6	0	0
3	1000	6	0	0
4	2000	6	0	0
5	4000	6	0	0
6	5000	6	1	13
7	6000	6	2	33.3

All deaths that occurred within 72 h of the extract administration were recorded and used to estimate the oral LD_50_ of the mice, which was found to be above 6000 mg/kg b.w. There were no differences between males and females regarding the symptoms and mortality at all doses. Postmortem macroscopic observations of both the survived and dead animals did not reveal any gross abnormality of the stomach, intestines, spleen, liver and kidneys.

### Chronic toxicity study

#### Effect of oral administration of GSG aqueous root extract on general health and body weight of the mice

Cage observations of the animals after administration of the extract at 400 and 800 mg/kg b.w were carried out daily for general signs of abnormalities throughout the study period. During the first week, the mice treated with 800 mg/kg b.w of the extract showed low locomotion, weakness, tremor (shivering) and piloerection. During the second week, these symptoms disappeared, and the animals completely recovered. After this, all animals in the treated and control groups appeared normal and did not show any extract-related clinical signs and symptoms of toxicity throughout the study period. No mortality was observed and all animals survived until the scheduled necropsy.

**Fig. 1:**
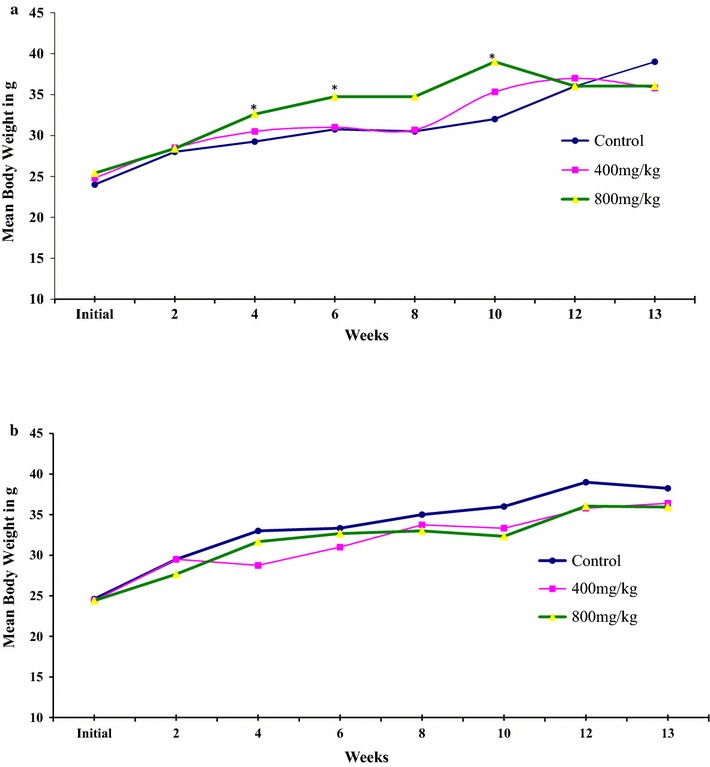
Effect of 400 and 800 mg/kg b.w/day of GSG root extract, administered for 13 weeks, on the body weight growth pattern of male (**a**) and female (**b**) mice. Each value point represents mean body weight; n = 5. *The mean difference is significant at a P value < 0.05

The body weight of each animal was recorded before the start of drug administration, once a week during drug administration and then at the end of the experiment at autopsy. The effect of GSG root extract on the body weight growth pattern of male and female mice during the 13 weeks of chronic treatment is shown in Fig. 1. Body weight of both treated and control groups rose progressively as the duration increased, and the extract did not show any significant effect on body growth patterns of the mice during the 13 weeks of chronic treatment. Besides, no significant difference was observed in the mean values of the body weights of male and female mice treated with 400 mg/kg b.w extract as compared to their respective controls. Treatment of male and female mice with 800 mg/kg b.w of the extract however, significantly increased body weight of male mice, but not that of female mice as compared to their respective controls. Moreover, there was no significant difference in the final body weight of all animals in the control and extract treated groups. However, significant increase in the mean values of the body weights of male mice treated with 800 mg/kg extract was observed during the fourth (P < 0.01), sixth (P < 0.05) and tenth (P < 0.01) weeks as compared to the mean body weights of the mice of both the control and 400 mg/kg treated groups.

#### Effect of GSG aqueous root extract on hematological parameters

The effect of 13 weeks chronic treatment with GSG root extract on hematological parameters of the mice is shown in Table 2. As indicated, chronic treatment with 400 mg/kg root extract did not significantly affect any of the investigated hematological parameters. Chronic treatment with 800 mg/kg extract however, induced significant (P < 0.05) rise in the RBC, HGB and in HCT-counts when compared to the control levels. Similarly, a significant (P < 0.05) increase in basophils count was observed with 800 mg/kg b.w extract. In contrast, lymphocytes count was significantly reduced with 800 mg/kg b.w GSG root extract as compared to the control.

**Table 2:** Effect of chronic oral administration of *G. stenophylla* root extract on some hematological parameters in mice

Hematological parameters	Control (DW)	Treatment groups (mg/kg body weight/day)	
		400	800
RBC (× 10^6^/µL)	3.44 ± 0.65	3.92 ± 0.66 (0.616)	6.04 ± 0.64 (0.040)*
HGB (g/dL)	5.35 ± 0.92	6.40 ± 1.11 (0.501)	9.48 ± 1.06 (0.042)*
HCT (%)	16.87 ± 3.28	19.23 ± 3.42 (0.627)	29.43 ± 3.05 (0.048)*
MCV (fL)	48.73 ± 1.44	48.77 ± 0.80 (0.976)	48.73 ± 0.12 (0.996)
MCH (pg)	15.70 ± 0.51	16.32 ± 0.28 (0.229)	115.70 ± 0.10 (1.00)
MCHC (g/dL)	32.20 ± 0.89	33.48 ± 0.37 (0.124)	32.20 ± 0.26 (1.00)
RDW (%)	17.98 ± 1.26	17.58 ± 0.74 (0.776)	20.17 ± 1.15 (0.198)
PLT (× 10^3^/µL)	215.25 ± 44.88	266.67 ± 49.12 (0.456)	315.33 ± 38.32 (0.231)
WBC (× 10^3^/µL)	6.02 ± 0.79	5.97 ± 0.79 (0.975)	4.89 ± 1.74 (0.483)
Neutrophils (%)	0.24 ± 0.09	0.44 ± 0.19 (0.565)	0.67 ± 0.48 (0.292)
Lymphocytes (%)	87.13 ± 5.58	92.20 ± 1.58 (0.507)	65.60 ± 11.94 (0.033)*
Monocytes (%)	2.73 ± 1.57	2.01 ± 0.91 (0.706)	2.16 ± 2.15 (0.796)
Eosinophils (%)	1.61 ± 0.65	1.25 ± 0.46 (0.646)	1.17 ± 0.63 (0.635)
Basophils (%)	7.79 ± 3.58	4.11 ± 0.57 (0.641)	30.40 ± 14.37 (0.031)*

Values are given as mean ± S.E.M. n = 10. The figures under brackets indicate the calculated P values of the treatment groups as compared to the control

*The mean difference is statistically significant at P value < 0.05

Moreover, GSG root extract treatment induced marginal increase in RBC count, HGB, and HCT with 400 mg/kg b.w of extract though not significant (Table 2). Extract treatment also showed a trend of dose dependant increment in PLT count and neutrophil percentage at the doses of 400 and 800 mg/kg b.w although not significant again. Monocytes and eosinophils, on the other hand, decreased non-significantly at both tested doses as compared to the control. In contrast to the above effects, there were no difference between values of MCV, MCH, MCHC, RDW and total WBC count of all GSG treated and control groups.

#### Effects of GSG root extract on serum biochemical parameters

Effects of chronic oral treatment with aqueous root extract of GSG on serum biochemical parameters of mice are shown in Table 3. All the parameters measured were not significantly different. Similarly, there was no significant difference in serum biochemical parameters of mice at 400 and 800 mg/kg b.w doses of extract (Table 3). Serum total protein level appeared to decrease after chronic treatment with 400 and 800 mg/kg of extract in a dose dependant manner. Treatment with the extract also appeared to decrease the serum ALP, ALT and urea levels at 400 and 800 mg/kg though non-significantly (Table 3). Dose-dependent changes were however, seen in ALT levels only. Treatment of animals with 400 and 800 mg/kg b.w of extract seem also to increase serum creatinine level. The two tested doses slightly increased serum AST level differently (Table 3). In all groups, serum cancer antigen-125 (CA-125) value was found to be in normal range, less than 0.02 IU/ml (Data not shown).

**Table 3:** Effect of chronic oral administration of *G. stenophylla* root extract on some serum biochemical parameters in mice

Biochemical parameters	Control	Treatment groups (mg/kg body weight/day)	
		400	800
Total protein (g/dL)	2.03 ± 0.05	1.96 ± 0.28 (0.842)	1.84 ± 0.21 (0.572)
ALP (IU/L)	50.00 ± 6.14	32.01 ± 15.72 (0.496)	47.00 ± 11.92 (0.909)
ALT (IU/L)	23.75 ± 0.85	22.60 ± 3.06 (0.777)	19.00 ± 3.05 (0.255)
AST (IU/L)	73.75 ± 12.66	87.80 ± 12.6 (0.520)	48.73 ± 0.12 (0.769)
Urea (mg/dL)	50.00 ± 3.42	38.80 ± 8.42 (0.281)	43.20 ± 6.44 (0.506)
Creatinine (mg/dL)	0.23 ± 0.03	0.30 ± 0.03 (0.144)	0.28 ± 0.04 (0.273)

Values are given as mean ± S.E.M. n = 10. The figures under brackets indicate the calculated P values of the treatment groups as compared to the control

#### Effects of GSG root extract on gross macroscopic appearance and wet organ weights

Postmortem macroscopic examination of all the studied internal organs, namely stomach, small intestine, large intestine, spleen, liver and kidneys for any possible changes in position, shape, size and color did not reveal any gross abnormality. Wet absolute weight (in g) and relative organ weight (in g per 100 g body weight) of liver, kidney and spleen of both extract treated and control groups are shown in Table 4. No significant difference was observed in absolute and relative organ weights of extract treated and control mice of either sex (Table 4). However, there was a slight, but not significant, decrease in absolute and relative weights of spleen at both doses in male mice, but not in females.

**Table 4:** Effect of chronic treatment with GSG root extract on the absolute and relative organ weights of liver, kidney and spleen of male and female mice as compared to the controls

	Control	Treatment groups (mg/kg body weight)	
		400	800
Liver			
Absolute weight (in g)			
Male	1.56 ± 0.07	1.48 ± 0.07 (0.630)	1.56 ± 0.14 (0.984)
Female	1.93 ± 0.02	1.78 ± 0.13 (0.396)	1.82 ± 0.16 (0.564)
Relative weight (in g/100 g bw)			
Male	3.92 ± 0.32	4.02 ± 0.24 (0.939)	4.25 ± 0.22 (0.575)
Female	5.04 ± 0.08	4.50 ± 0.16 (0.344)	5.02 ± 0.51 (0.998)
Kidney			
Absolute weight (in g)			
Male	0.26 ± 0.01	0.25 ± 0.01 (0.410)	0.25 ± 0.06 (0.597)
Female	0.21 ± 0.01	0.19 ± 0.01 (0.444)	0.22 ± 0.02 (0.362)
Relative weight (in g/100 g bw)			
Male	0.66 ± 0.06	0.69 ± 0.03 (0.900)	0.68 ± 0.04 (0.936)
Female	0.54 ± 0.02	0.50 ± 0.02 (0.667)	0.62 ± 06 (0.233)
Spleen			
Absolute weight (in g)			
Male	0.14 ± 0.02	0.11 ± 0.01 (0.100)	0.12 ± 0.01 (0.100)
Female	0.17 ± 0.01	0.16 ± 0.02 (0.639)	0.18 ± 0.02 (0.731)
Relative weight (in g/100 g bw)			
Male	0.36 ± 0.07	0.30 ± 0.01 (0.390)	0.32 ± 0.01 (0.630)
Female	0.44 ± 0.03	0.43 ± 0.07 (0.990)	0.49 ± 0.05 (0.755)

Values are given as mean ± S.E.M. n = 10. The figures under parenthesis indicate the calculated P values of the treatment groups as compared to the control. *Bw* body weight

#### Effect of aqueous root extract of GSG on histopathology of the spleen

Histological examinations of sections of the spleen from mice treated with the extract with 400 mg/kg b.w was found normal revealing lymphoid masses of white pulp, and a highly vascular red pulp as compared to spleens of control mice, which showed the normal histology (data not shown, but provided as an Additional file 1). As shown in the additional file stated, spleen histology of mice treated with 800 mg/kg was also normal and no abnormality was observed. In both treated and control mice the white pulp consisted of an irregular mass of lymphoid tissues in the vicinity of an arteriole, as splenic follicles. The follicles exhibited germinal centers and a narrow mantle zone at periphery beyond which is a broader marginal zone. Besides, erythrocytes and lymphocytes pool, the red pulp contained a number of hemosiderin-containing macrophages and megakaryocytes which are the constant normal constituents of normal mice spleen.

#### Effects of GSG aqueous root extract on histopathology of the liver

**Fig. 2:**
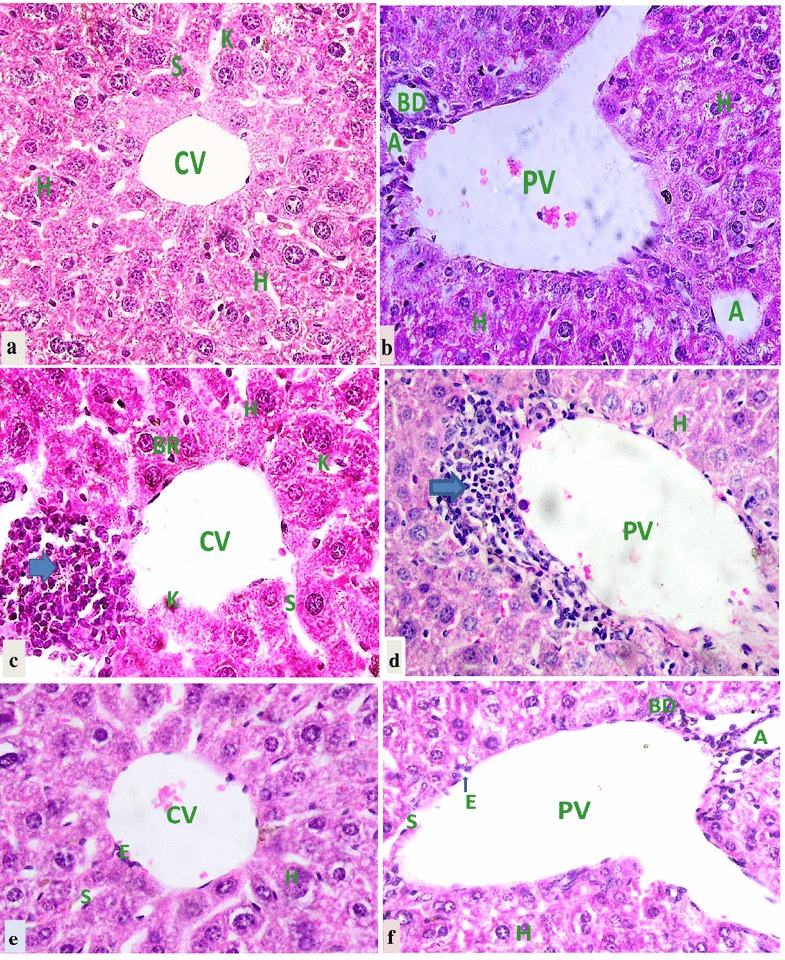
Photomicrographs of H and E stained liver sections of mice treated with 400 (**a** and **b**) and 800 mg/kg body weight (**c** and **d**) of GSG root extract and liver sections of control mice (**e** and **f**). Inflammatory cellular infiltrations (arrows) around central vein (cv) and portal vein (pv) were seen in the mice treated 800 mg/kg body weight of extract (**c** and **d**). A in **b** and **f** terminal branch of hepatic artery, BD in **b** and **f** bile duct, CV in **a**, **c** and **f** central vein, H in all hepatocytes, K in **a** and **c** *Kupffer* cells, PV in **b**, **d** and **f** portal vein, S in **a**, **c**, **e** and **f** hepatic sinusoid. Magnifications, all ×4200

Histological examinations of liver sections of mice treated with 400 mg/kg b.w extract (Fig. 2a and b) showed a normal architecture with normal appearance of the central vein and hepatic sinusoids lined with endothelial and *Kupffer* cells similar to the controls (Fig. 2e and f). The hepatocytes appeared normal in size and shape, and no vacuoles were noted in their cytoplasm. In the mice treated with 800 mg/kg b.w (Fig. 2c and d), however, there was intrahepatic (as a yellowish discoloring of the hepatocytes) and extrahepatic (as deposits in the bile canaliculi between the individual hepatocytes) bile retention indicating induction of cholestasis (biliary stasis). Moreover, central veins and portal triads were infiltrated and expanded by mononuclear leukocytic cells (Fig. 2c and d), indicating mild hepatitis. On the other hand, the normal lobular architecture of the liver was not affected; hepatocytes and *Kupffer* cells also appeared normal. Besides, there was no treatment-related change in the diameter and appearance of central veins, hepatic sinusoids and portal veins.

#### Effects of GSG aqueous root extract on histopathology of the kidneys

Histopathological evaluation of kidney sections of mice treated with the aqueous extract of GSG roots for 13 weeks at oral doses of 400 and 800 mg/kg b.w indicated no histopathological changes as compared to that of the controls. Data showing this result was provided as an Additional file 2. Renal histology of both control and extract treated animals exhibited normal features: intact glomerular and tubular structures. The capillary tufts and size of the Bowman’s space also appeared normal. Moreover, the proximal convoluted tubules, distal convoluted tubules, Henle’s loop, collecting tubules, macula densa, and mesangial cells appeared normal after administration of both doses as compared to control kidneys.

## Discussion

The oral LD_50_ of the extract was found to be above 6000 mg/kg body weight in mice, suggesting that the lethal dose is far (more than ten times) greater than the effective antimalarial dose (i.e. 400 mg/kg b.w). This result is in agreement with the result of the previous study [23]. Absence of gross pathological findings in those animals died with 5000 and 6000 mg/kg b.w extract needs further scientific explanation for the mechanism of death, but it might be due to death from functional disorder rather than structural abnormality.

Chronic treatment in this study also indicated that the extract was well tolerated by all animals, as no mortality and extract-related signs and symptoms of toxicity were observed in either sex throughout the study period. The extract had no harmful effect on body growth patterns of test groups. The significant increase in body weights of the male mice treated with 800 mg/kg observed during the fourth, sixth and tenth weeks might not be considered treatment related as no similar increment was observed in female mice and the changes did not show dose–response pattern. In line with this, no data was available regarding the effects of GSG on the body weight of animals and/or humans [28, 29].

The role of blood as an index of pathological and physiological status in humans and animals is well documented [35–38]. In acute and chronic toxicological studies, changes in hematological as well as biochemical parameters are usually used as indices of toxicities. Measurement of RBC count, HCT and HGB can be used to determine anemia, which could be due to a decrease in the total number of erythrocytes, or reduced red blood cell size (MCV), or reduced hemoglobin amount per erythrocyte (MCH), or diminished concentration of hemoglobin per total erythrocytes (MCHC) [39, 40].

In the present study, oral administration of 400 or 800 mg/kg b.w of GSG root extract for 13 weeks did not decrease the levels of all red blood cell indices (MCV, MCH and MCHC), total RBC count, HCT and HGB, indicating that it does not cause anemia. Instead, elevations in the levels of RBC count, HCT and HGB in a dose-dependent pattern were observed, which might be due the potential of the plant extract on activation of erythropoiesis. Treatment with 800 mg/kg b.w of the extract also revealed significant elevation of basophils, which might be indicative of basophilia at high dose. On the contrary, graded oral administration of the extract lowered the monocyte and eosinophil differentials in a dose related fashion. This suggests that the extract could have either selective toxicity for monocyte and eosinophil lineages or may induce vascular margination of these granulocytes. Besides, administration of 800 mg/kg b.w of extract decreased the percentage of lymphocytes significantly, indicating induction of lymphocytopaenia which is consistent with earlier reports [25] on the lymphocytopaenic effects of *G. latifolia* in bull calves. The lymphocytes were significantly decreased in number in response to stressful condition after antigen (extract) entrance as reported by Moussa [35] and Adedapo et al. [36]. Since lymphocytes play the key role in all immune reactions [41], they migrate to sites of inflammations, while their number may concomitantly decrease from circulation in the blood as were observed in the present study at the higher dose.

Toxic agents are known to affect the liver, kidney, GIT, spleen, and other organs and impair their physiological functions. Due to the cardinal role of the kidneys in excreting waste products like blood urea and creatinine, and their ability to filter and reabsorb the body-needed threshold substance like electrolytes, the levels of these serum biochemical parameters could be used as renal function tests [38, 39]. Such measurement can also give an insight to the site of cellular tissue damage because of assault by the potential toxic agent on repeated exposure.

The nonclinical safety study recommendations for plant products that have special cause for concern or intended for a long duration of use may also include assessment of carcinogenic potential. Serum CA-125 and CA-99.5 levels are commonly used to assess carcinogenic potential of drugs. In accordance with these principles, levels of serum total protein, ALP, ALT and AST were measured in the present study and used as liver function tests, while urea and creatinine levels were used as kidney function tests [42–45]. Similarly, serum CA-125 level was used to assess any carcinogenic potential of GSG aqueous root extract in mice.

The biological rationale behind analyzing both body weight and organ weight to body weight ratio (relative organ weight) is that in the majority of cases, except for brain, the organ weight change is proportional to total body weight. Hence, they are normally investigated to determine whether the size of organ has changed, particularly in relation to the weight of the whole animal as indicator of adverse effect of chemicals on that organ. It is for this reason that this study investigated the effect of chronic treatment of GSG root extract on absolute and relative organ weights as well as on gross and histopathological parameters of spleen, liver and kidneys. Treatment with different doses of the extract did not produce any detectable and meaningful change in mean absolute or relative organ weights of liver, kidney and spleen in male and female mice. Changes in weights of some organs was not considered treatment related because no similar decrement was found in either sex and the changes did not show dose–response pattern. To our knowledge, no scientific evidence has ever adduced the effects of *Gnidia* species on organ weights of animals.

Extract related toxicity might cause changes in indicators of immunotoxicity such as hematological parameters of leukocytes and histopathology of lymphoid organs such as the spleen [25]. However, no difference was seen in total leukocytes count and serum total protein in the control and treated groups following chronic administration of the extract at both doses. Besides, no differences in the absolute and relative weights of spleen were detected between control and treated groups. On the other hand, the extract significantly decreased the lymphocytes percentage and induced lymphocytopaenia with 800 mg/kg b.w of the extract. However, this significant difference was not accompanied by any histopathological change in spleen, as microscopic evaluation of spleen sections of extract treated mice appeared normal as compared to those of control mice. This result is however in contrary to the earlier reports [25], which described lymphocytic degeneration and cellular depletion in the follicles of lymph nodes and spleen of bull calves fed *G. latifolia.* The difference might be due to variation in phytochemical composition of the two related *Gnidia* species.

Liver being the major target organ of toxicity, injury to the liver may affect the integrity of hepatocytes leading to the release of membrane bound enzymes (e.g. ALT and AST), damage to hepato-biliary system thereby causing the release of essential enzymes (e.g. ALP), and/or impair the biosynthetic and catabolic capacity of the liver. It can also show histopathological abnormalities, such as hepatocyte degenerative changes and fat accumulation [42–45]. Oral administration of GSG aqueous root extract at 400 or 800 mg/kg b.w for 13 weeks in the current study, did not affect the absolute and relative weights of liver in both sexes. Besides, gross macroscopic evaluation of liver did not show any lesion at both doses in either sex in comparison to control livers. No significant differences in biochemical markers of liver toxicity (serum total protein, ALP, ALT and AST levels) between the control and treated groups were observed. Histopathological evaluation indicated that liver sections of mice treated with 800 mg/kg b.w showed some intrahepatic (as a yellowish discoloring of the hepatocytes) and extrahepatic (as deposits in the bile canaliculi between the individual hepatocytes) cholestasis (biliary stasis). Inflammatory cells infiltrations were also seen around the central vein and portal tract area.

The slight histopathological changes observed in mice of the high dose group were not accompanied by any significant change in the biochemical markers of measured liver injury. Furthermore, fatty change, hydropic degeneration, fibrosis, and vascular abnormalities such as congestion of the central veins and liver sinusoids usually seen in hepatotoxicity [45–48] were not seen in any of the treated groups. Therefore, the aqueous root extract of GSG can be considered nonhepatotoxic and have minimal adverse effect on the liver of the mice at the tested doses. The biochemical, gross and histopathological findings obtained in the liver in this study are in line with those of Kiptoon et al. [25], who described the absence of any significant change in liver function tests (AST, ALP and ALT) and in liver sections of high grade bull calves fed *G. latifolia*.

No significant differences in absolute and relative weights of the kidneys was observed in the control and extract treated mice at both dose levels. None of the values of the kidney function tests measured (serum urea and creatinine levels) were significantly different in the treated and control animals. Besides, histological examination comparison of the kidney sections of the treated mice and control mice revealed no significant changes attributable to treatment with the extract. In all groups, the renal glomerular and tubular components were intact and appeared normal. Moreover, none of the signs of glomerulonephritis [45–47] was observed in any of the mice treated with the extract. Therefore, the aqueous root extract of GSG appears to have no toxicity to the kidney.

## Conclusions

The results of this safety evaluation and toxicological investigation of *G. stenophylla* Gilg showed that the tested aqueous crude extract of the plant at an antimalarial dose (400 mg/kg b.w/day) had no serious adverse effect on body growth, organ weights, and hematological and biochemical parameters as well as on gross and histopathological appearance of spleen, liver and kidneys in mice. Moreover, *G. stenophylla* Gilg does not have carcinogenic potential. Higher dose (800 mg/kg b.w/day) of the extract may have a potential of increasing some hematological indices such as RBC, HCT, HGB and platelets count up on prolonged administration, it, however, may induce irritation of liver tissues as a side effect. Further investigation on other vital organs and non-rodent species, including human is recommended since mild hepatotoxicity was observed on prolonged administration. Nevertheless, absence of any morbidity as well as any significant sign of toxicity even at high dose up to 5000 mg/kg b.w appears to support the general safety of *G. stenophylla* Gilg aqueous root extract for treatment of malaria and may contribute towards the development of new antimalarial drug from the species.

## Additional files

**Additional file 1.** Histomicrograph of spleen.

**Additional file 2.** Histomicrograph of kidney.

## Abbreviations

AAU: Addis Ababa University
ALP: alkaline phosphatase
ALT: alanine aminotransferase
AST: aspartate aminotransferase
b.w: body weight
CA-125: cancer antigen-125
cm: centimeter
dl: deciliter
DW: distilled water
EDTA: ethylene diamine tetra-acetic acid
EPHI: Ethiopia Public Health Institute
fL: femtoliter (10^−15^ L)
g: gram
GSG: *Gnidia stenophylla* Gilg
HCT: hematocrit
H&E: hematotoxylin-eosin stain
HGB: hemoglobin
IU: international unit
Kg: kilogram
LD_50_: lethal dose for 50-percent of the population
m: meter
µ: micron
µL: microliter
mbr: milibar
MCV: mean corpuscular volume
MCH: mean corpuscular hemoglobin
MCHC: mean corpuscular hemoglobin concentration
mg: milligram
N(n): sample size (number of mice)
Pg: pictogram (10^−12^g)
PLT: platelet count
RBC: red blood cells
RDW: red cell distribution width
S.E.M: standard error of mean
SPSS: statistical package support solution
TMMDRD: Traditional and Modern Medicine, Drug Research Directorate
WBC: white blood cells
WHO: World Health Organization
