# Supplementary material for: Toxicological investigation of acute and chronic treatment with Gnidia stenophylla Gilg root extract on some blood parameters and histopathology of spleen, liver and kidney in mice
Source: BMC Res Notes. 2017 Nov 28;10:625. doi: 10.1186/s13104-017-2964-3 (PMC5704563; doi:10.1186/s13104-017-2964-3)
Supplement: Supplementary file 1 — Additional file 1. Histomicrograph of spleen. [file 13104_2017_2964_MOESM1_ESM.docx]

**b**

**a**

**d**

**c**

**

**e**

**f**

**Additional file 1:** Photomicrographs of H and E stained spleen sections of mice treated with 400 **(a** and **b)** and 800 mg/kg body weight/day **(c** and **d)** of GSG aqueous root extract for 13 weeks showing normal histology of the white pulp **(WP)** and red pulp (**RP)** areas as compared to that of the controls (**e** and **f**). **GC** in **a, c & e=** germinal center**, WP** in **a, c & e=** white pulp**, RP** in **b**, **d & f=** red pulp**, MnZ** in **a, c & e=** mantle zone**, MgZ** in **a, c & e=** marginal zone**, A** in **a=** arteriole**, HM** in **b, d & f=** hemosiderin-containing macrophages, **MK** in **b, d & f**= megakaryocytes. Magnifications, all= x4200.
