# Supplementary material for: Toxicological investigation of acute and chronic treatment with Gnidia stenophylla Gilg root extract on some blood parameters and histopathology of spleen, liver and kidney in mice
Source: BMC Res Notes. 2017 Nov 28;10:625. doi: 10.1186/s13104-017-2964-3 (PMC5704563; doi:10.1186/s13104-017-2964-3)
Supplement: Supplementary file 2 — Additional file 2. Histomicrograph of kidney. [file 13104_2017_2964_MOESM2_ESM.docx]

**Additional file 2:** Photomicrographs of **H** and **E** stained kidney sections of mice treated with aqueous root extract of GSG at 400 mg/kg (**a**) and at 800 mg/kg b.w/day **(b)** showing no histopathological changes as compared to that of the control group **(c**). **BS** in all= *Bowman’s* space*,* **DCT** in all= distal convoluted tubule, **G** in all**=** glomerulus, **MD** in **a**= macula densa, **PCT** in all= proximal convoluted tubule, **UP** in **a**= urinary pole. Magnifications, all= X4200.

**a**

**b**

**c**
